# Antiviral Treatment among Older Adults Hospitalized with Influenza, 2006-2012

**DOI:** 10.1371/journal.pone.0121952

**Published:** 2015-03-25

**Authors:** Mary Louise Lindegren, Marie R. Griffin, John V. Williams, Kathryn M. Edwards, Yuwei Zhu, Ed Mitchel, Alicia M. Fry, William Schaffner, H. Keipp Talbot

**Affiliations:** 1 Department of Pediatrics, Vanderbilt University School of Medicine, Nashville, Tennessee, United States of America; 2 Department of Medicine, Vanderbilt University School of Medicine, Nashville, Tennessee, United States of America; 3 Department of Health Policy, Vanderbilt University School of Medicine, Nashville, Tennessee, United States of America; 4 Mid-South Geriatric Research Education and Clinical Center, VA TN Valley Health Care System, Nashville, Tennessee, United States of America; 5 Department of Pathology, Microbiology, and Immunology, Vanderbilt University School of Medicine, Nashville, Tennessee, United States of America; 6 Vanderbilt Vaccine Research Program, Department of Pediatrics, Vanderbilt University School of Medicine, Nashville, Tennessee, United States of America; 7 Department of Biostatistics, Vanderbilt University School of Medicine, Nashville, Tennessee, United States of America; 8 Centers for Disease Control and Prevention, Atlanta, Georgia, United States of America; Faculty of Biochemistry Biophysics and Biotechnology, Jagiellonian University, POLAND

## Abstract

**Objective:**

To describe antiviral use among older, hospitalized adults during six influenza seasons (2006—2012) in Davidson County, Tennessee, USA.

**Methods:**

Among adults ≥50 years old hospitalized with symptoms of respiratory illness or non-localizing fever, we collected information on provider-initiated influenza testing and nasal/throat swabs for influenza by RT-PCR in a research laboratory, and calculated the proportion treated with antivirals.

**Results:**

We enrolled 1753 adults hospitalized with acute respiratory illness. Only 26% (457/1753) of enrolled patients had provider-initiated influenza testing. Thirty-eight patients had a positive clinical laboratory test, representing 2.2% of total patients and 8.3% of tested patients. Among the 38 subjects with clinical laboratory-confirmed influenza, 26.3% received antivirals compared to only 4.5% of those with negative clinical influenza tests and 0.7% of those not tested (p<0.001). There were 125 (7.1%) patients who tested positive for influenza in the research laboratory. Of those with research laboratory-confirmed influenza, 0.9%, 2.7%, and 2.8% received antivirals (p=.046) during pre-pandemic, pandemic, and post-pandemic influenza seasons, respectively. Both research laboratory-confirmed influenza (adjusted odds ratio [AOR] 3.04 95%CI 1.26-7.35) and clinical laboratory-confirmed influenza (AOR 3.05, 95%CI 1.07-8.71) were independently associated with antiviral treatment. Severity of disease, presence of a high-risk condition, and symptom duration were not associated with antiviral use.

**Conclusions:**

In urban Tennessee, antiviral use was low in patients recognized to have influenza by the provider as well as those unrecognized to have influenza. The use of antivirals remained low despite recommendations to treat all hospitalized patients with confirmed or suspected influenza.

## Introduction

Influenza is estimated to cause an average of 200,000 hospitalizations and 3,300 to 49,000 deaths each year in the US.[1–4] Since the 2009–2010 H1N1 influenza pandemic, the Centers for Disease Control and Prevention (CDC) has recommended prompt use of antiviral treatment for all hospitalized patients with confirmed or suspected influenza. [5, 6] Use of antiviral treatment among hospitalized patients has been associated with reduced mortality, with earlier treatment resulting in better outcomes. [6–8] Despite these recommendations, barriers to prompt antiviral treatment among hospitalized patients include lack of reliable rapid influenza diagnostic tests, late presentation of patients to care, difficulty distinguishing influenza clinically from other acute respiratory infections and a lack of confidence in the effectiveness of antivirals.[9–11] Additionally, influenza often manifests atypically in adults ≥50 [12, 13], presenting as exacerbations of underlying conditions such as asthma or chronic obstructive pulmonary disease (COPD). Few data are available on trends in the use of antiviral therapy among high-risk, hospitalized, older adult populations.

We described the use of antivirals among adults 50 years of age and older who were hospitalized with symptoms of acute respiratory illness or non-localizing fever over six influenza seasons from 2006–2012 in Davidson County, Tennessee. We analyzed how often influenza was tested for and diagnosed by the treating providers, what methods were used, and the frequency of antiviral treatment. We also independently tested all participants for influenza using RT-PCR in a research laboratory as part of influenza vaccine effectiveness studies, regardless of clinical testing.[14–18] We further examined predictors of antiviral treatment, including demographics, duration of symptoms at the time of hospitalization, underlying chronic conditions, results from clinical testing, year of influenza season, diagnosis of pneumonia, and indicators of disease severity (as defined by ICU admission, intubation, and/or new oxygen requirement).

## Methods

### Study Description

Over six consecutive years, adults ≥50 years hospitalized with symptoms of acute respiratory illness or non-localizing fever at four hospitals in Davidson County, Tennessee (Nashville and environs) were enrolled from November 2006 through April 2012. Two of these hospitals conducted surveillance in the first two influenza seasons and four hospitals from 2008 onward. Analyses were restricted to patients that presented during influenza season, defined as the period encompassing all identified influenza infections in the research laboratory at Vanderbilt University Medical Center. During the 2009 pandemic, surveillance continued from spring 2009 through the spring of 2010 to capture the entire pandemic period. At enrollment, information was collected on comorbid medical conditions, self-reported influenza vaccination status, smoking history, and use of certain medications (i.e., chemotherapy, immunosuppressive medications, steroids, and antiviral medications).

### Study Population

Patients’ ≥50 years of age hospitalized with the following admission diagnoses (*International Classification of Diseases, 9^th^ Revision* Number) were enrolled: pneumonia (480–486), upper respiratory infection (465), bronchitis (466), influenza (487), chronic obstructive pulmonary disease (490 to 492; 496), asthma (493), viral illness (079.9), dyspnea (786), acute respiratory failure (518.81), pneumonitis due to solids/liquids (507), or fever (780.6) without localizing symptoms. Eligible presenting symptoms included cough, non-localizing fever, shortness of breath, sore throat, and nasal congestion or coryza. [1, 2]

### Study Design

At the time of enrollment, the subject or caretaker was asked a series of brief questions about the present illness, smoking history, and influenza vaccination. Medical records were reviewed to obtain information on demographic data, past medical history, antiviral medication use, results of microbiologic and radiographic tests, and types and results of provider-initiated influenza diagnostic studies using a standardized form.

### Laboratory Methods

Research testing: nasal and throat swabs were obtained, placed in viral media (Remel M4RT), and tested in the research laboratory for influenza virus by real-time reverse-transcriptase polymerase chain reaction (RT-PCR) as previously described.[14] [15] Influenza positive cases were defined as subjects with positive RT-PCR on duplicate testing. Samples were tested by primers and probes specific for influenza A and influenza B. If positive for influenza A, samples were then tested for H1N1 and H3N2 subtyping. Subjects were defined as research laboratory influenza negative if the nose/throat sample tested negative for influenza by initial RT-PCR testing and had evidence of β-actin or RNaseP, indicating human cells in the sample. Remaining samples were defined as indeterminate and were excluded from the analyses.

### Definitions

Physician initiated clinical diagnostic laboratory testing was available for those subjects whose treating provider ordered testing from the clinical laboratory of their respective hospital, and included rapid antigen detection, PCR, or viral culture. Results of positive RT-PCR tests from the research laboratory were not available to treating providers. Influenza seasons were defined by the total number of weeks that included all influenza positive specimens in both the research and clinical laboratories at Vanderbilt University Medical Center in Nashville, TN each year. (S1 Fig.) Antiviral use was defined as in-hospital receipt of a neuraminidase inhibitor (oseltamivir, zanamivir), adamantane (amantadine, rimantadine) or experimental antiviral agent (IV peramivir, IV zanamivir) by chart review.

### Analysis

We compared characteristics of antiviral treated and untreated subjects among three groups: all subjects, those with clinical laboratory-confirmed influenza, and those with research laboratory-confirmed influenza. Chi-square or Fisher’s exact tests were used as appropriate to compare antiviral-treated and -untreated groups.

Pre-specified explanatory variables for antiviral treatment included the number of days from symptom onset to hospitalization, underlying high-risk conditions (defined as transplant, cancer within 5 years, diabetes, asplenia, heart or vascular disease, kidney or liver disease, sickle cell disease, asthma or chronic bronchitis or COPD or other lung disease, memory or thinking problems, HIV/AIDS or other problems with the immune system, genetic or metabolic disorders, neurologic disease, currently on prednisone or other steroids, any chemotherapy in the last 6 months, or any immunosuppressive medications in the last 6 months), age, influenza season, gender, self-reported vaccination status, discharge diagnosis of influenza or pneumonia, severity of illness (combination of oxygen received during hospital stay, ICU admission or required intubation) and results of influenza testing performed by both the clinical and research laboratory.

Univariate and multivariable logistic regression models evaluated the association between antiviral treatment and potential predictive factors. After testing for collinearity, importance of covariates, and consideration of the convention of maintaining at least 10 outcomes (subjects who received antiviral treatment) per degree of freedom to minimize the risk of model over-fitting[19], we included clinical laboratory influenza test status, research laboratory influenza status, and discharge diagnosis of influenza or pneumonia in our final model for all study subjects and for the subgroup of subjects with research laboratory-confirmed influenza. The clinical laboratory influenza test status explanatory variable was selected for the univariate logistic regression model. The correlation between research and clinical laboratory influenza status was assessed. A large proportion (74%) of patients did not have clinical laboratory tests for influenza performed in the hospital. There was significant disagreement between research and clinical laboratory tests (McNemar test p< 0.001). Therefore, both clinical and research testing were analyzed as potential risk factors for antiviral treatment in the model, although research laboratory influenza results were not available to clinicians when antiviral treatment decisions were made.

Adjusted odds ratios (AOR) for antiviral treatment and 95% confidence intervals were calculated for each predictive factor in the two groups of laboratory testing. All analysis were done using R version 2.15.1.

### Ethics

This study protocol was approved by the Institutional Review Board of Vanderbilt University Medical Center. Eligible subjects or their legally authorized representative provided written informed consent. In rare instances, when a patient was unable to consent for his/her self and family/legal advocate was not available, the institutional review board approved a waiver of consent.

## Results

### Patient Characteristics

From 2006 to 2012, a total of 4020 adults ≥ 50 years hospitalized with symptoms of acute respiratory illness or fever were eligible for the study, of whom 2071 (51.5%) consented to participate; 1761 were hospitalized while influenza was circulating in the community, and 1753 had an adequate nasal/throat sample and a valid research laboratory RT-PCR test result. Those who refused participation were similar to those who participated by gender (58% female vs. 56% female) and race (white race 81% vs. 81%). However, a larger proportion of those who refused were aged ≥65 years (69%) compared to participants (56%; p<0.001).

Of the 1753 hospitalized patients, 56% were aged ≥65 years, 78% were white, 21% black, 93% had at least one underlying high-risk condition, and 68% reported receiving an influenza vaccination that season. (Table 1) Fifty-three percent of enrolled subjects had symptoms for ≥3 days prior to hospitalization.

**Table 1:** Characteristics of Adults 50 years and older Hospitalized with Symptoms of Respiratory Illness or non-localizing Fever during 2006–2012 Influenza Season.

Characteristic	All patients	Patients with research laboratory- confirmed influenza	Patients with clinical laboratory- confirmed influenza	P-value (research vs. clinical)
	N = 1753	N = 125	N = 38	
	N (%)	N (%)	N (%)	
Gender				
Female	981 (56)	75 (60)	22 (58)	
Male	772 (44)	50 (40)	16 (42)	0.82
Age group, years				
50–64	778 (44)	72 (58)	25 (66)	
65+	975 (56)	53 (42)	13 (34)	0.37
Race				
White	1361 (78)	90 (72)	25 (66)	
Black	362 (21)	33 (26)	13 (34)	
Other	18 (1)	2 (2)	0 (0)	
Unknown	12 (<1)	0 (0)	0 (0)	0.44
High-Risk Condition*				
No	125 (7)	16 (13)	7 (18)	
Yes	1628 (93)	109 (87)	31 (82)	0.38
Required oxygen during hospitalization/ intubation during hospitalization/ admission to the ICU				
No	419 (24)	27 (22)	10 (26)	
Yes	1334 (76)	98 (78)	28 (74)	0.54
Duration of illness before hospitalization, days				
0d or missing	318 (18)	12 (10)	2 (5)	
1-2d	511 (29)	34 (27)	10 (26)	
> = 3d	924 (53)	79 (63)	26 (68)	0.68
Any Insurance				
No	60 (3)	9 (7)	2 (5)	
Yes	1651 (94)	110 (88)	35 (92)	
Unknown	42 (2)	6 (5)	1 (3)	0.75
Self-reported influenza vaccination status				
No	480 (27)	58 (46)	17 (45)	
Yes	1204 (69)	62 (50)	20 (53)	
Unknown	69 (4)	5 (4)	1 (3)	0.94
Influenza testing for research		—-		
Negative	1628 (93)	—-	11 (29)	
Positive	125 (7)	125	27 (71)	—
Provider Flu testing during hospitalization				
Negative	419 (24)	40 (32)	—-	
Positive	38 (2)	27 (22)	38	
Not Done	1296 (74)	58 (46)	—-	—
Discharge Diagnosis of Influenza or Pneumonia				
No	1060 (61)	64 (51)	7 (18)	
Yes	693 (39)	61 (49)	31 (82)	<0.01
Discharge Diagnosis of Circulatory or Respiratory Disease				
No	143 (8)	6 (5)	1 (3)	
Yes	1610 (92)	119 (95)	37 (97)	1.0

*defined as transplant, cancer within 5 years, diabetes, no spleen, heart or vascular disease, kidney or liver disease, sickle cell disease, asthma or chronic bronchitis or COPD or other lung disease, memory or thinking problems, HIV/AIDS or other problems with the immune system, genetic or metabolic disorders, neurologic disease, currently on prednisone or other steroids, any chemotherapy in the last 6 months, or any immunosuppressive medications in the last 6 months.

### Influenza Testing

**Fig 1:**
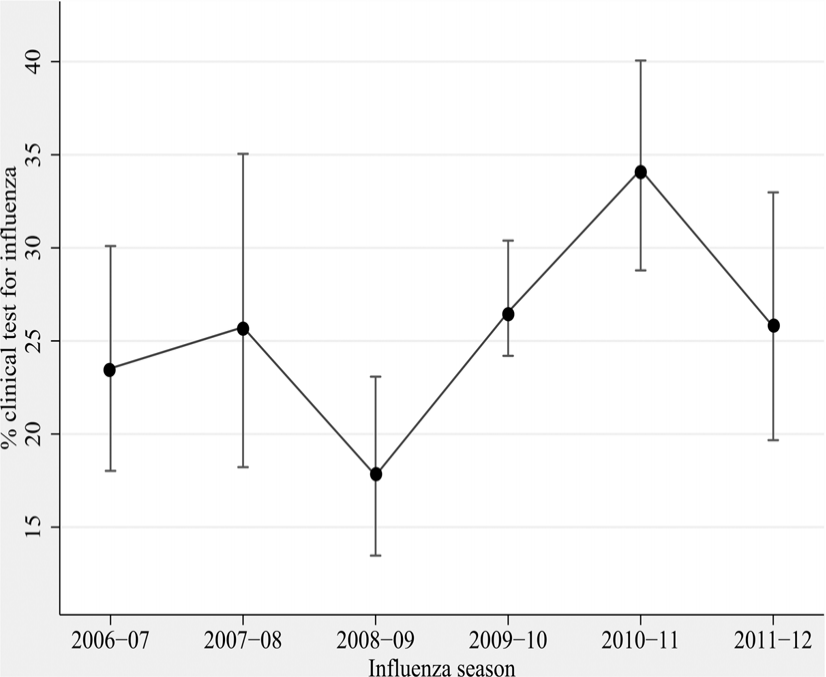
Clinical diagnostic testing among adults ≥50 years of age hospitalized with acute respiratory illness, by season, 2006–2012.

Overall, physician initiated influenza testing was performed in the clinical laboratory, as ordered by the treating physician, in 457 of the 1753 subjects (26%), averaging 21.3% during pre-pandemic years to 26.6% during pandemic to 31.0% during post-pandemic years (p<.001). (Fig. 1) Testing varied significantly between the four study hospitals, ranging from 10.8% to 39.0% of patients enrolled, p<0.01. Testing varied over the influenza season, but the only significant difference in proportion tested by month occurred during pandemic when 51.8% of enrolled patients were tested during September 2009 (data not shown). The annual proportion of clinical laboratory tests that were positive ranged from 4.6% in 2006–2007 to 16.7% in 2011–2012. Of 399 patients who had a rapid influenza test performed, 4.8% (N = 19) were positive; of the 58 who had a clinical laboratory PCR or viral culture performed, 32.8% (N = 19) were positive.

There were 125 (7.1%) of 1753 patients with influenza detected by RT-PCR in the research laboratory, ranging from 3.2% during the 2009–2010 pandemic to 18.8% during the 2007–2008 influenza season. Of those with research laboratory-confirmed influenza, 54% (67/125) also had a clinical laboratory test performed. Of the 53 (80%) with a rapid test only 25% (13/53) were positive. All research laboratory positive samples tested in the clinical laboratory by PCR or culture were positive (100%; 14/14).

### Antiviral treatment

Overall, only 38 of 1753 (2.2%) patients hospitalized with symptoms of acute respiratory illness or fever during the six influenza seasons received antivirals, including 10 of 38 (26.3%) patients with clinical laboratory-confirmed influenza and 14 of 125 (11.2%) patients with research laboratory-confirmed influenza. (Table 2) The only antiviral administered was oseltamivir. There were no differences in antiviral treatment by sex, race, underlying high-risk condition, duration of symptoms, self-report of influenza vaccine, admission to the ICU, or requiring intubation or oxygen during hospitalization.

**Table 2:** Characteristics of Adults 50 years and older Hospitalized with Symptoms of Respiratory Illness or Non-localizing fever during 2006–2012 Influenza Season, by Antiviral Treatment Status.

	All patients				Patients with research laboratory-confirmed influenza				Patients with clinical laboratory-confirmed Influenza			
Characteristic	Total	Did Not Receive Antiviral Treatment	Received Antiviral Treatment	P value	Total	Did Not Receive Antiviral Treatment	Received Antiviral Treatment	P value	Total	Did Not Receive Antiviral Treatment	Received Antiviral Treatment	P value**
	N	N (%)	N (%)		N	N (%)	N (%)		N	N (%)	N (%)	
All	1753	1715 (97.8)	38 (2.2)		125	111 (88.8)	14 (11.2)		38	28 (73.7)	10 (26.3)	
Gender												
Female	981	959 (97.8)	22 (2.2)	0.808	75	68 (90.7)	7 (9.3)	0.418	22	17 (77.3)	5 (22.7)	0.71
Male	772	756 (97.9)	16 (2.1)		50	43 (86.0)	7 (14.0)		16	11 (68.8)	5 (31.3)	
Age group, years												
50–64	778	755 (97.0)	23 (3.0)	0.043	72	64 (88.9)	8 (11.1)	0.971	25	19 (76.0)	6 (24.0)	0.71
65+	975	960 (98.5)	15 (1.5)		53	47 (88.7)	6 (11.3)		13	9 (69.2)	4 (30.8)	
Race												
White	1361	1328 (97.6)	33 (2.4)	0.309	90	78 (86.7)	12 (13.3)	0.059	25	16 (64.0)	9 (36.0)	0.12
Black	362	358 (98.9)	4 (1.1)		33	32 (97.0)	1 (3.0)		13	12 (92.3)	1 (7.7)	
Other	18	17 (94.4)	1 (5.6)		2	1 (50.0)	1 (50.0)		0	0 (0)	0 (0)	
Unknown	12	12 (100.0)	0 (0)		0	0 (0)	0 (0)		0	0 (0)	0 (0)	
High-risk condition*												
No	125	120 (96.0)	5 (4.0)	0.187	16	13 (81.3)	3 (18.8)	0.388	7	4 (57.1)	3 (42.9)	0.35
Yes	1628	1595 (98.0)	33 (2.0)		109	98 (89.9)	11 (10.1)		31	24 (77.4)	7 (22.6)	
Required ICU admission,												
oxygen, or intubation												
No	419	407 (97.0)	12 (3.0)	0.253	27	24 (89.0)	3 (11.0)	1	10	7 (70.0)	3 (30.0)	1.0
Yes	1334	1308 (98.0)	26 (2.0)		98	87 (89.0)	11 (11.0)		28	21 (75.0)	7 (25.0)	
Duration of illness												
before hospitalization (days)												
0d or missing	318	315 (99.1)	3 (0.9)	0.061	12	10 (83.3)	2 (16.7)	0.473	2	2 (100.0)	0 (0)	0.55
1-2d	511	503 (98.4)	8 (1.6)		34	32 (94.1)	2 (5.9)		10	8 (80.0)	2 (20.0)	
> = 3d	924	897 (97.1)	27 (2.9)		79	69 (87.3)	10 (12.7)		26	18 (69.2)	8 (30.8)	
Any Insurance												
No	60	57 (95.0)	3 (5.0)	0.2	9	7 (77.8)	2 (22.2)	0.393	2	1 (50)	1 (50)	0.62
Yes	1651	1616 (97.9)	35 (2.1)		110	98 (89.1)	12 (10.9)		35	26 (74.3)	9 (25.7)	
Unknown	42	42 (100.0)	0 (0)		6	6 (100)	0 (0)		1	1 (100)	0 (0)	
Self-reported influenza												
vaccination status												
No	480	465 (96.9)	15 (3.1)	0.137	58	50 (86.2)	8 (13.8)	0.558	17	12 (70.6)	5 (29.4)	0.80
Yes	1204	1181 (98.1)	23 (1.9)		62	56 (90.3)	6 (9.7)		20	15 (75.0)	5 (25.0)	
Unknown	69	69 (100.0)	0 (0)		5	5 (100.0)	0 (0)		1	1 (100.0)	0 (0)	
Influenza testing for research												
Negative	1628	1604 (98.5)	24 (1.5)	<0.001		==			11	10 (90.9)	1 (9.1)	0.22
Positive	125	111 (88.8)	14 (11.2)			==			27	18 (66.7)	9 (33.3)	
Provider influenza testing												
during hospitalization												
Negative	419	400 (95.5)	19 (4.5)	<0.001	40	36 (90.0)	4 (10.0)	<0.001		—		
Positive	38	28 (73.7)	10 (26.3)		27	18 (66.7)	9 (33.3)			—		
Not Done	1296	1287 (99.3)	9 (0.7)		58	57 (98.3)	1 (1.7)			—		
Discharge diagnosis of												
influenza or pneumonia												
No	1060	1047 (98.8)	13 (1.2)	0.001	64	62 (96.9)	2 (3.1)	0.003	7	6 (85.7)	1 (14.3)	0.65
Yes	693	668 (96.4)	25 (3.6)		61	49 (80.3)	12 (19.7)		31	22 (71.0)	9 (29.0)	
Discharge diagnosis of												
circulatory or respiratory disease												
No	143	142 (99.3)	1 (0.7)	0.362	6	6 (100.0)	0 (0)	1	1	1 (100.0)	0 (0)	1.0
Yes	1610	1573 (97.7)	37 (2.3)		119	105 (88.2)	14 (11.8)		37	27 (73.0)	10 (27.0)	

*defined as transplant, cancer within 5 years, diabetes, no spleen, heart or vascular disease, kidney or liver disease, sickle cell disease, asthma or chronic bronchitis or COPD or other lung disease, memory or thinking problems, HIV/AIDS or other problems with the immune system, genetic or metabolic disorders, neurologic disease, currently on prednisone or other steroids, any chemotherapy in the last 6 months, or any immunosuppressive medications in the last 6 months.

** Chi-square or Fisher’s exact tests were used where appropriate to compare antiviral treated and untreated groups.

#### All Study Participants

**Fig 2:**
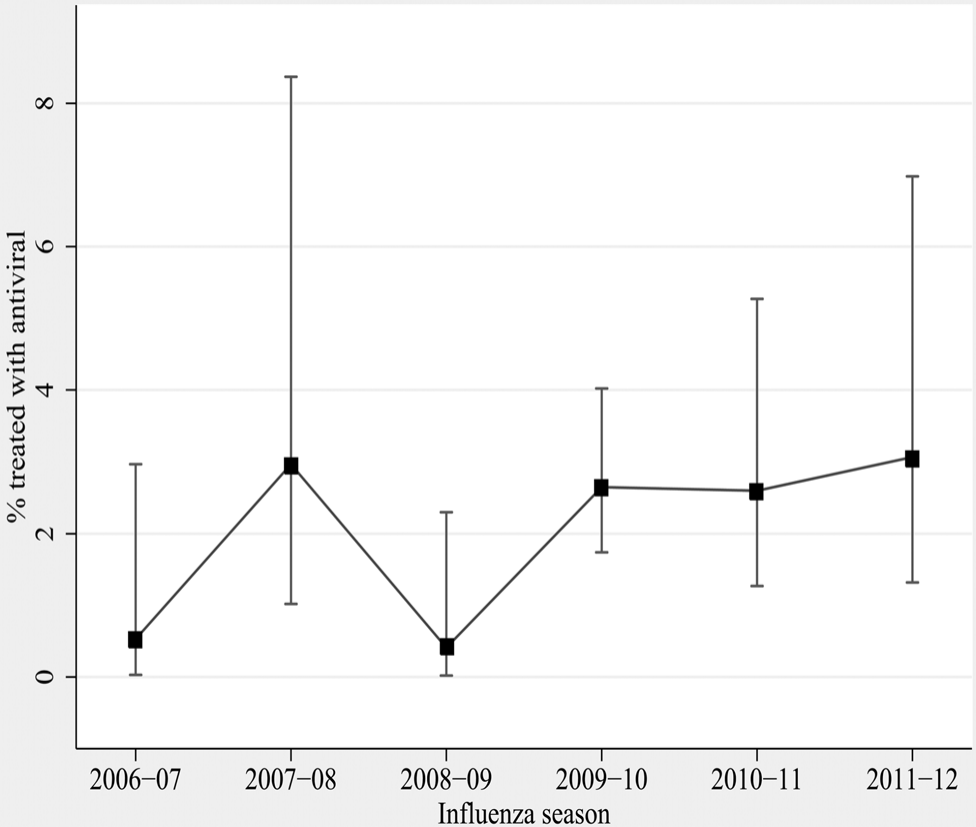
Antiviral treatment among adults ≥50 years of age hospitalized with acute respiratory illness, by season, 2006–2012.

Overall, the proportion of 1753 patients who received antivirals was low (2.2%), ranging from 0.5%-3.0% annually (Table 3) (Fig. 2). Although antiviral use increased from 0.9% during pre-pandemic years to 2.7% (N = 21) during pandemic and to 2.8% (N = 12) in post-pandemic years (p = 0.046), use continued to be low. (Table 3) Of those hospitalized patients prescribed antivirals, 87% (33/38) had underlying high-risk conditions. (Table 2) Approximately 29% (511/1753) of patients were hospitalized within two days of symptom onset, yet antiviral use was low in this group as well (1.6%) (8/511). Treatment rates varied between the four study hospitals, ranging from 0.6% (2/344) to 2.4% (11/467).

**Table 3:** Trends in Use of Antiviral Treatment—2006–2012.

Influenza Season	All patients			Patients with research laboratory- confirmed influenza			Patients with clinical laboratory- confirmed influenza tests		
	Did not receive antivirals	Received antivirals	% Antiviral Treatment	Did not receive antivirals	Received antivirals	% Antiviral Treatment	Did not receive antivirals	Received antivirals	% Antiviral Treatment
2006–2007	186	1	0.5	13	1	7.1	2	0	0.0
2007–2008	98	3	3.0	16	3	15.8	1	3	75.0
2008–2009	241	1	0.4	9	1	10.0	3	0	0.0
2009–2010*	770	21	2.7	20	5	20.0	10	5	33.3
2010–2011	262	7	2.6	38	2	5.0	7	0	0.0
2011–2012	158	5	3.1	15	2	11.8	5	2	28.6
Total	1715	38	2.2	111	14	11.2	28	10	26.3

*Pandemic season.

Antiviral treatment was more common in those age 50–64 years compared to 65 years and older (3.0% (23/778) vs. 1.5% (15/974)), p = 0.04; those with a discharge diagnosis of influenza or pneumonia compared to those with other discharge diagnoses [3.6% (25/693) vs. 1.2% (13/1060), p = 0.001]; those with versus those without research laboratory-confirmed influenza [11.2% (14/125) vs. 1.5% (24/1628), p<0.001]; and those with a positive vs. negative clinical laboratory influenza test [26.3% (10/38) vs. 4.5% (19/419)] or no test [0.7% (9/1296), p<0.001]. Of those 38 patients who received antiviral treatment, nine (23.7%) had no clinical laboratory test for influenza.

#### Clinical Laboratory-confirmed influenza

Of 38 patients with clinical laboratory-confirmed influenza, 26.3% (N = 10) received antiviral treatment compared to 4.5% (N = 19) of 419 patients with a negative clinical test and 0.7% (N = 9) of 1296 patients who were not clinically tested for influenza (p<0.001).

Antiviral treatment of those with clinical laboratory-confirmed influenza ranged from 0 of 2 (0%) in 2006–2007 to 3 of 4 (75%) in 2007–2008, and 5 of 15 (33%) during the 2009–2010 pandemic. The numbers in each year were too small to assess trends. (Table 3) In this small group, there were no significant differences between those who received antivirals and those who did not. (Table 2)

#### Research Laboratory-confirmed influenza

Among the 125 patients with research laboratory-confirmed influenza, antiviral use increased from 11.6% during pre-pandemic years to 20% during pandemic, but declined to 7% during post pandemic years (p = ns). (Table 3) Similar to results for the entire study population, patients who received antivirals vs. those who did not were more likely to have a positive provider ordered influenza test (33%) vs. a negative (10%) or no test (1.7%), p<0.001, and have a discharge diagnosis of pneumonia or influenza (19.7% vs. 3.1%, p = 0.003). (Table 2) Among those with laboratory-confirmed influenza, 81% were influenza A and 19% were Influenza B; treatment rates were similar for those with influenza A (12%), compared to B (8%); p = 0.74

#### Factors associated with antiviral treatment in multivariate analysis

In the analysis that included all patients, research laboratory-confirmed influenza (AOR 3.04, 95% CI: 1.26–7.35) and clinical laboratory-confirmed influenza compared to negative clinical test results (AOR 3.05, 95% CI: 1.07–8.71) were positively associated with antiviral treatment; and lack of clinical influenza testing was negatively associated with antiviral treatment (AOR 0.17, 95% CI: 0.07–0.38) (Table 4). A discharge diagnosis of influenza or pneumonia was not associated with antiviral treatment.

**Table 4:** Independent factors associated with antiviral treatment among adults, 50 years and older, hospitalized with symptoms of acute respiratory illness or non-localizing fever, and those with research laboratory-confirmed influenza, 2006–2012.

Factor	All patients^1^ (N = 1753)	Patients with research laboratory-confirmed influenza ^2^(N = 125)
	Adjusted Odds ratio (95% CI)	Adjusted Odds ratio (95% CI)
Clinical laboratory influenza testing		
Negative	Referent	Referent
Positive	3.05 (1.07–8.71)	4.50 (1.22, 16.62)
Not done	0.17 (0.07, 0.38)	0.16 (0.02, 1.47)
Discharge diagnosis of pneumonia/influenza	1.92 (0.93, 3.97)	————
Research laboratory- confirmed influenza	3.04 (1.26, 7.35)	————

^1^ For all patients, the variables included in the model included 1) clinical laboratory influenza testing, 2) research laboratory influenza status, and 3) discharge diagnosis of influenza or pneumonia.

^2^ For patient with research laboratory-confirmed patients, the variable included clinical laboratory influenza tests status.

In the analysis of patients with research laboratory-confirmed influenza, only one variable, clinical laboratory influenza testing was included in the final model. Clinical laboratory-confirmed influenza compared to negative clinical test results was associated with receipt of antiviral treatment (AOR 4.5, 95% CI: 1.22, 16.62). Those who had no clinical influenza testing were less likely to receive antivirals than those with negative tests (AOR 0.16, 95% CI: 0.02, 1.47).

## Discussion

Over six influenza seasons (2006–2012) in urban Tennessee, use of antiviral treatment was low among hospitalized patients ≥50 years recognized to have influenza by their provider and those unrecognized to have influenza. The use of antivirals remained low despite recommendations to treat all hospitalized patients with confirmed or suspected influenza and changed little over time. Clinical testing for influenza remained infrequent and consisted primarily of rapid antigen tests, which have a low sensitivity in our study population (25%)[20]. Nevertheless, confirmed influenza by clinical laboratory testing was a predictor of antiviral treatment. Research laboratory testing (not available to the clinicians) was also a predictor of treatment and identified many more patients with influenza who could have benefited from treatment with antivirals. Persons who were treated in the absence of a positive clinical test were much more likely to be influenza positive by research testing than untreated patients, indicating that for a small subset of subjects, clinicians correctly identified those likely to have influenza. Those subjects who had no clinical laboratory testing performed, the population eligible for empiric treatment, were less likely to receive antivirals than those with negative test results, suggesting that providers tested those patients with a perceived higher likelihood of having influenza. However, despite positive clinical testing associated with receipt of antivirals, the number of subjects receiving antivirals still remained low. Strikingly, receipt of antivirals was not independently associated with severity of illness, duration of symptoms, underlying high-risk conditions, influenza season, or age.

Since 2009, the CDC, the Advisory Committee on Immunization Practices (ACIP), and the Infectious Disease Society of America (IDSA) have recommended empiric antiviral treatment for patients who are hospitalized with confirmed or suspected influenza. [6, 21] Although no randomized-controlled trials have specifically evaluated the effectiveness of antiviral treatment among hospitalized patients, supportive evidence comes from observational studies. Recent systematic reviews of observational studies among high-risk populations report reduced mortality among those who received oseltamivir. [7] Earlier treatment was generally associated with better outcomes.[7, 8] However, more effectiveness studies of antivirals on patient important clinical outcomes among high-risk populations are needed, especially among those presenting for care beyond 48 hours after the onset of illness.[22]

In our study, clinicians infrequently prescribed antivirals to hospitalized patients at high-risk for morbidity and mortality. Suboptimal use of antivirals among hospitalized patients has been reported in other studies, with declining use since the pandemic. [23–27] There was very low antiviral use (2%) reported in a similar study of young children hospitalized with acute respiratory infection and laboratory-confirmed influenza (2004–2009) in three sites of the National Vaccine Study Network (NVSN) network, including Davidson County, TN.[28] Antiviral use among hospitalized adults with physician-ordered, laboratory-confirmed influenza identified through active population-based surveillance in the Emerging Infections Program (EIP) in 10 states, increased from 54% before and to 82% during the 2009 pandemic and then declined to 6% among adults in the 2010–2011 season. [23, 24] Factors associated with receipt of antiviral treatment were a positive rapid influenza diagnostic test and being hospitalized ≤ 2 days after illness onset. In a population of adults who self-reported influenza-like illness, sought care, and received a diagnosis of influenza, 36% reported receiving antiviral drugs during the 2009 pandemic. [29] Use of antivirals was low based on US hospital discharge data during the pandemic, where one third of those hospitalized did not receive antivirals.[30] These studies have also found that antiviral treatment use was higher among those with positive rapid tests. [25, 29] Evaluating patient populations with positive physician-ordered influenza tests compared to a broader population of patients with acute respiratory symptoms likely overestimates of antiviral coverage and does not include the entire population that may benefit from testing or treatment. However, our estimate of antiviral coverage among patients who had positive physician-ordered tests (26%), was similar to other studies, but was much lower among the broader yet eligible hospitalized patients with symptoms of acute respiratory illness (2%).

Availability of accurate and timely diagnostic tests represents a potential challenge to use of antivirals. Overall, use of influenza diagnostic testing among hospitalized patients was low and most of the provider-ordered tests were rapid tests. Point-of-care rapid influenza diagnostic tests have high specificity (>90%) but low to moderate sensitivity (20–70%) compared to RT-PCR, with much lower sensitivity among adults compared to children.[6, 20, 31] Due to limited sensitivity, negative rapid tests results are not useful in making decisions about antiviral treatment, particularly among older adults. RT-PCR testing is more sensitive and specific for detecting influenza viruses; however its use has been limited by availability, costs, and turn-around time.[6, 32] More accurate point-of-care influenza tests that are readily available to providers in a timely manner could facilitate receipt of antivirals among hospitalized patients.

Timely diagnosis of influenza is important to reduce use of antibiotics, reduce need for additional diagnostic testing, and increase use of early antiviral treatment [14, 21, 33–35] thereby reducing unnecessary and indiscriminant use of antimicrobials in viral illness. A recent study among outpatients with PCR-confirmed influenza reported infrequent use of antivirals by clinicians, even among those at high-risk for complications, but antibiotics were prescribed at a higher rate than antivirals, most of which were likely unnecessary.[27] Our findings emphasize the need for clinician education on use of antivirals for patients hospitalized with suspected influenza, and may impact potential overuse of antibiotics.

Despite increasing evidence of cost-effectiveness of antiviral treatment among high-risk populations, [32, 36, 37], including several studies that found any antiviral treatment is more cost effective than no treatment, challenges exist to implementation. Surveys of physicians indicate that antivirals are often not prescribed due to late presentation to clinical care, cost of antivirals, uncertain diagnosis of influenza, and concern about antiviral drug effectiveness. [9, 10] Clinical symptoms of respiratory illness caused by influenza are similar to those caused by other respiratory pathogens, making a clinical diagnosis challenging. [11] However, the most significant predictor of antiviral use in our study was a positive test that was ordered by the health care provider, suggesting providers were testing patients who were more likely to have influenza. Clinical judgment, including disease severity, clinical presentation, and experience are important factors in treatment decisions among providers. [38, 39] This may likely explain why clinical testing was a predictor of receipt of antivirals in our study, despite overall low clinical testing.

Several limitations exist for this study. We did not enroll all hospitalized patients with influenza or influenza-like illness. Enrolled adults differed from non-enrolled adults in age and possibly other unmeasured characteristics. Our data are representative of Davidson County regarding use of viral testing and antiviral use, but these could vary geographically outside this area. Finally, we included self-reported influenza vaccination status as a possible predictor of antiviral use as opposed to confirmation of vaccination status from providers and registries as this is likely the only information available to clinicians when a patient is hospitalized.

## Conclusion

Despite recommendations for early, universal use of antivirals for all hospitalized patients with confirmed or suspected influenza since the H1N1 pandemic (2009–2010) antiviral use was low. Although there was a moderate increase in influenza testing since the pandemic, testing remained low and used mostly rapid antigen tests, which we and others have shown to be insensitive for detecting influenza in hospitalized adults ≥50 years. Antiviral treatment without a positive influenza test was rare. These results confirm that antivirals sometimes are not used because influenza is not clinically diagnosed, due to the poor sensitivity of the rapid influenza tests. More accurate point of care influenza tests could facilitate receipt of antivirals among hospitalized patients. Because treatment of hospitalized patients with antivirals is associated with reductions in morbidity or mortality, CDC and IDSA guidelines recommend use of antivirals for all hospitalized patients with confirmed or suspected influenza. Additional strategies are needed to improve appropriate antiviral treatment among hospitalized adults with influenza, particularly for older adults with severe disease or with underlying high-risk conditions.

## Supporting Information

S1 Fig Influenza activity by CDC week and year. (TIF)
