# Supplementary figures and images for: Antiviral Treatment among Older Adults Hospitalized with Influenza, 2006-2012
Source: PLoS One. 2015 Mar 25;10(3):e0121952. doi: 10.1371/journal.pone.0121952 (PMC4373943; doi:10.1371/journal.pone.0121952)

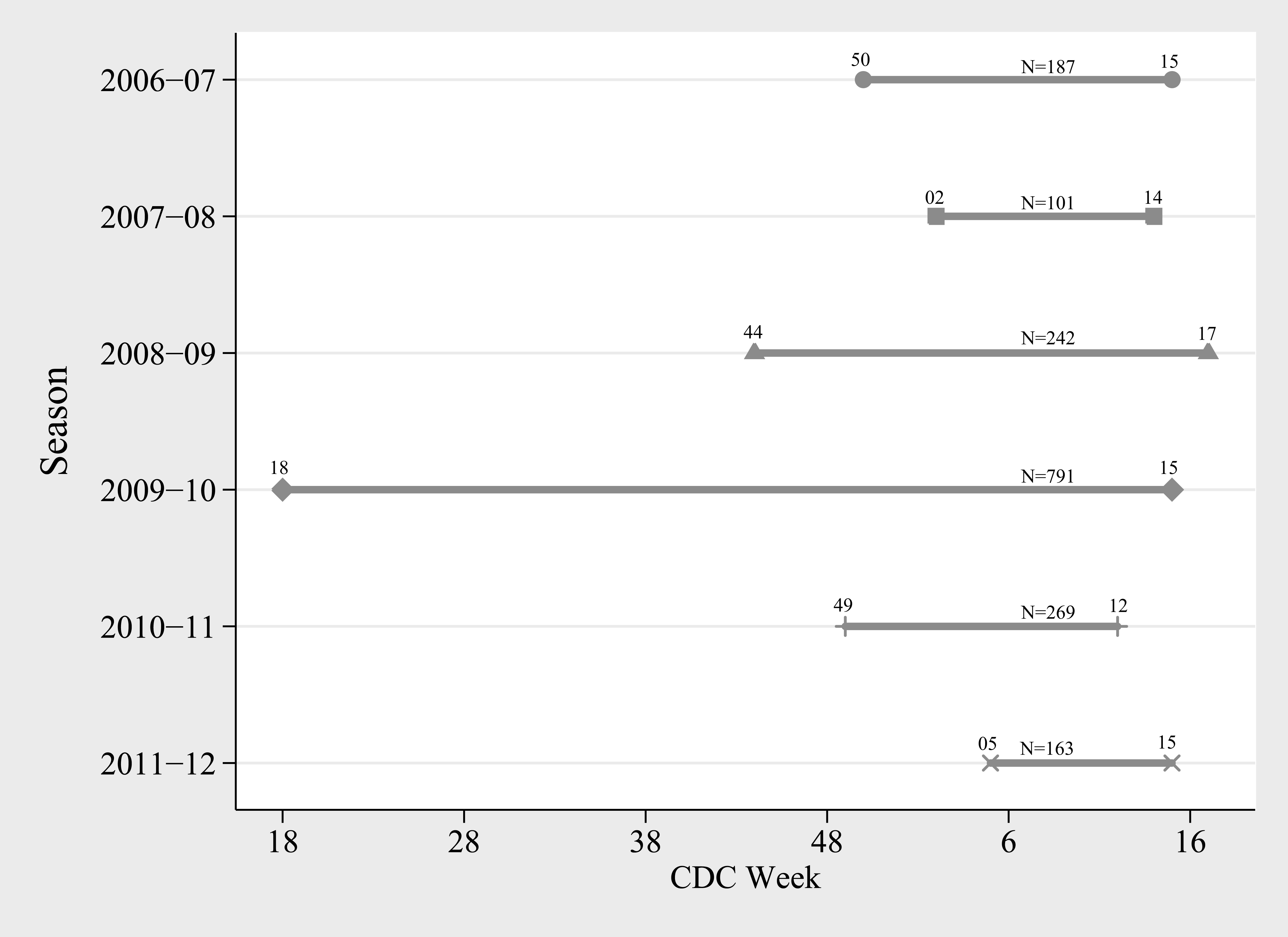

Supplement: S1 Fig — (TIF) [file pone.0121952.s001.tif]
